# Selective β-Mono-Glycosylation of a C15-Hydroxylated Metabolite of the Agricultural Herbicide Cinmethylin Using Leloir Glycosyltransferases

**DOI:** 10.1021/acs.jafc.1c01321

**Published:** 2021-05-11

**Authors:** Jihye Jung, Katharina Schmölzer, Doreen Schachtschabel, Michael Speitling, Bernd Nidetzky

**Affiliations:** † Austrian Centre of Industrial Biotechnology, Graz A-8010, Austria; ‡ BASF SE, Carl-Bosch-Strasse 38, Ludwigschafen 67056, Germany; § Institute of Biotechnology and Biochemical Engineering, NAWI Graz, TU Graz, Graz A-8010, Austria

**Keywords:** cinmethylin, Leloir glycosyltransferase, glycosylation, xenobiotic metabolism, agricultural herbicide

## Abstract

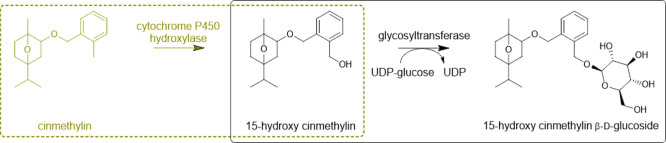

Cinmethylin is a well-known benzyl-ether derivative of the natural terpene 1,4-cineole that is used industrially as a pre-emergence herbicide in grass weed control for crop protection. Cinmethylin detoxification in plants has not been reported, but in animals, it prominently involves hydroxylation at the benzylic C15 methyl group. Here, we show enzymatic β-glycosylation of synthetic 15-hydroxy-cinmethylin to prepare a putative phase II detoxification metabolite of the cinmethylin in plants. We examined eight Leloir glycosyltransferases for reactivity with 15-hydroxy cinmethylin and revealed the selective formation of 15-hydroxy cinmethylin β-d-glucoside from uridine 5′-diphosphate (UDP)-glucose by the UGT71E5 from safflower (*Carthamus tinctorius*). The UGT71E5 showed a specific activity of 431 mU/mg, about 300-fold higher than that of apple (*Malus domestica*) UGT71A15 that also performed the desired 15-hydroxy cinmethylin mono-glycosylation. Bacterial glycosyltransferases (OleD from *Streptomyces antibioticus*, 2.9 mU/mg; GT1 from *Bacillus cereus*, 60 mU/mg) produced mixtures of 15-hydroxy cinmethylin mono- and disaccharide glycosides. Using UDP-glucose recycling with sucrose synthase, 15-hydroxy cinmethylin conversion with UGT71E5 efficiently provided the β-mono-glucoside (≥95% yield; ∼9 mM) suitable for biological studies.

## Introduction

**Figure 1:**
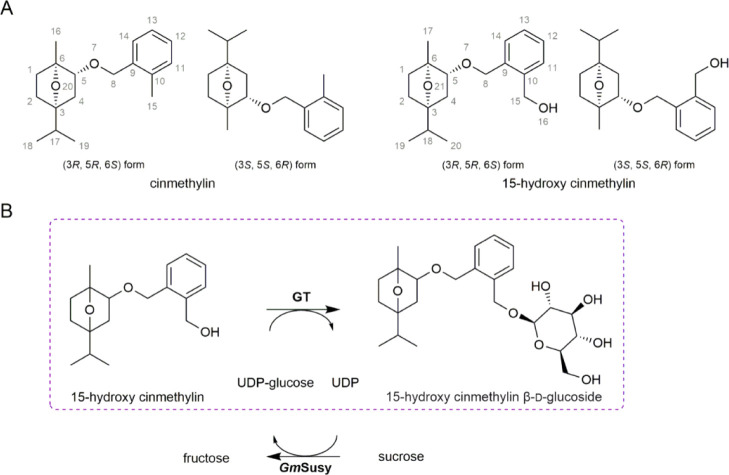
Cinmethylin and enzymatic glycosylation of 15-hydroxy cinmethylin. (A) Cinmethylin and its C15-hydroxylated derivative of cinmethylin (15-hydroxy cinmethylin) with atoms numbered. The 15-hydroxy cinmethylin used is a 1:1 mixture of the isomers shown. (B) Glycosylation of 15-hydroxy cinmethylin by GT and UDP-glucose. The product is 15-hydroxy cinmethylin β-d-glucoside. The UDP-glucose substrate can be regenerated from sucrose and UDP by sucrose synthase, here from soybean (Glycine max), abbreviated GmSusy.

Plant protection chemicals represent a rapidly growing sector within the agrochemical industries. Among these chemicals, herbicides are important active substances to control weed growth. Approval procedures for herbicides require detailed evaluation of environmental safety.^1−4^ The assessment of the fate and behavior in the environment entails that not only the original substances but also their detoxification metabolites are closely monitored.^3,5,6^ Cinmethylin (Figure 1) is a well-known benzyl-ether derivative of the natural terpene 1,4-cineole.^7−9^ Cinmethylin is used industrially as a pre-emergence herbicide for crop protection.^7−10^ Commercialized cinmethylin-based products are marketed for integrated grass weed management (*e.g.*, Luximax and Luximo) to provide control against various grasses (*e.g.*, ryegrass and blackgrass) with developed resistance(s). Cinmethylin is classified into group Z of the herbicide mode of action groups, implying interference with the cellular membrane structure and function due to the inhibition of fatty acid thioesterases by cinmethylin-derived 1,4-cineole.^7^ Metabolic studies in animals (*e.g.*, rat and goat) support the idea that phase I detoxification of cinmethylin involves single- and multiple-site hydroxylation events.^11,12^ Among the cinmethylin metabolites found in goats, the benzylic C15 methyl group of cinmethylin was a prominent site for hydroxylation.^12^ Indirect evidence from cinmethylin efficacy studies in plants supports a role of cytochrome P450 hydroxylases in compound detoxification.^9^ Further (phase II) detoxification of hydroxylated cinmethylin metabolites would likely involve glycosylation, with a β-d-glucosyl residue attached in plants^3,5,6,13^ and a β-d-glucuronyl residue attached in animals.^14,15^ The current study was performed to develop a biocatalytic synthesis for the β-d-glucoside of 15-hydroxy cinmethylin (Figure 1). The 15-hydroxy cinmethylin was synthesized chemically. The resulting 15-hydroxy cinmethylin β-d-glucoside was of considerable interest to facilitate later biological studies of cinmethylin metabolism associated with cinmethylin in planta efficacy. Although chemical glycosylation of 15-hydroxy cinmethylin would be possible using general methodology,^16^ an enzymatic route that enables selective β-glucosylation of unprotected reactants in a flexibly scaled, single-step transformation is highly desirable and has not been described.

To prepare the 15-hydroxy cinmethylin-β-d-glucoside (Figure 1), we first considered whether to embark on glycosylation by glycoside hydrolases/*trans*-glycosidases,^17−21^ glycoside phosphorylases,^22−25^ or sugar nucleotide-dependent (Leloir) glycosyltransferases (GTs).^26−31^ Each enzyme type could in principle be used to glycosylate 15-hydroxy cinmethylin. However, small-molecule glycosylation in detoxification metabolism is naturally performed by GTs.^6,13^ In plants, these enzymes use uridine 5′-diphosphate (UDP)-glucose as the sugar donor.^26,27^ Substrate requirements of the GTs are sometimes adduced to favor enzymes from other classes working with more expedient substrates.^26−28^ No natural GT for the glycosylation of 15-hydroxy cinmethylin is known. Our search for a candidate enzyme for synthesis took into account two criterions in particular. The enzyme’s reaction selectivity should be high. Due to their intrinsic hydrolase activity, glycoside hydrolases/*trans*-glycosidases often fail this criterion.^22,26^ The enzyme’s specificity for the acceptor substrate should be relaxed, to accommodate the “non-physiological” 15-hydroxy cinmethylin with suitable reactivity. Glycoside phosphorylases usually prefer carbohydrate acceptors.^22−25^ Therefore, a broadly specific catalyst applicable to precision synthesis of 15-hydroxy cinmethylin β-d-glucoside was likely to be found among the Leloir GTs.

Here, we therefore examined eight permissive GTs, known from previous studies to exhibit broad tolerance for the structure of the acceptor substrate in the reaction with UDP-glucose, for reactivity with 15-hydroxy cinmethylin (Figure 1). The set of GTs selected was representative rather than exhaustive of enzymes from the literature. Besides permissive reactivity with the acceptor substrate, practical use of the GT (*e.g.*, recombinant production in *Escherichia coli*; specific activity; and stability) was considered. Four GTs were from plants: UGT71E5 from safflower (*Carthamus tinctorius*),^32^ UGT71A15 from apple (*Malus domestica*),^33,34^ Indian snake root (*Rauvolfia serpentina*) arbutin synthase,^33,35^ and maize (*Zea mays*) UGT708A6.^33^ Three GTs were from bacteria: *Bacillus cereus* GT1^36,37^ and *Streptomyces antibioticus* OleD in the wildtype and triple variant form.^38,39^ A human glucuronyltransferase from xenobiotic metabolism^15,40,41^ was additionally tested. The enzymes were expressed in *E. coli*, purified, and confirmed to be active in standard glycosylation reactions. Kinetic assays for glycosylation of 15-hydroxy cinmethylin from UDP-glucose were performed and UGT71E5 was identified from the enzyme screening. The UGT71E5 reaction was developed for preparative synthesis of 15-hydroxy cinmethylin β-d-glucoside in a selective manner. Sucrose synthase (from soybean; *Glycine max*)^42,43^ was used in a coupled reaction with UGT71E5 (Figure 1) for the synthesis of 15-hydroxy cinmethylin β-d-glucoside from sucrose in the presence of catalytic concentration of UDP. The UDP-glucose regeneration thus established eliminates concern about the use of a sugar nucleotide donor for glycosylation of 15-hydroxy cinmethylin. The study is novel for its identification of UGT71E5 for β-glycosylation of 15-hydroxy cinmethylin and the development of a biocatalytic synthesis of the target β-d-glucoside in high yield.

## Materials and Methods

### Chemicals

Chemicals were from Carl Roth (Karlsruhe, Germany) and Sigma-Aldrich (Vienna, Austria) in highest purity available. UDP disodium salt, UDP-d-glucose disodium salt, UDP-d-glucuronic acid ammonium salt, 7-amino-4-methylcoumarin, and phloretin (98%) were from Carbosynth (Berkshire, UK). 4-Methylumbelliferone (≥98%) was from Sigma-Aldrich.

### Chemical Synthesis of 15-Hydroxy Cinmethylin

The 15-hydroxy cinmethylin (Figure 1) was synthesized chemically at BASF, using a generally known route.^44,45^ Like the commercial cinmethylin (Figure 1), 15-hydroxy cinmethylin is a mixture of (3*R*, 5*R*, and 6*S*) and (3*S*, 5*S*, and 6*R*) enantiomers. An isomer ratio of 1:1 was shown for the used 15-hydroxy cinmethylin by an optical rotation of ∼0°, measured at 589 nm and 20 °C with a PerkinElmer 341 polarimeter (Waltham, MA, USA). The 15-hydroxy cinmethylin used had a high-performance liquid chromatography (HPLC)-estimated purity of ≥99.5%. The 15-hydroxy cinmethylin is toxic and irritant. Proper care was taken in its handling.

### Enzymes

Synthetic genes optimized for expression in *E. coli* were from BioCat GmbH (Heidelberg, Germany). The following constructs were used considering the literature on the respective enzyme. *Bc*GT1 (origin: *B. cereus*; GenBank accession number: KT821092; no tag);^37^ OleD wildtype (origin: *S. antibioticus*; GenBank accession number: DQ195536.2; also referred to as UGT102A2; *N*-terminal His tag);^38,39^ OleD triple variant ASP (A242V/S132F/P167T; *N*-terminal His tag);^38,39^ UGT71E5 (origin: *C. tinctorius*; GenBank accession number: KX610759.1; *N*-terminal His tag);^32^ UGT1A9 (origin: Homo sapiens; GenBank accession number: AF056188.1; *N*-terminal maltose binding protein; C-terminal His tag);^46^ arbutin synthase (origin: *R. serpentina*; GenBank accession number: CAC35167.1; *N*-terminal Strep tag);^33,35^ UGT71A15 (origin: *M. domestica*; GenBank accession number: DQ103712; *N*-terminal Strep tag),^34^ and UGT708A6 (origin: *Z. mays*; GenBank accession number: ACF81582.1; *N*-terminal Strep tag)^33,47^ were obtained as described recently.

Plasmid vectors and *E. coli* expression strains are described in the Supporting Information. Enzyme production was done under standard conditions (Supporting Information) with expression by isopropyl-β-d-thiogalactoside at lowered temperature (18–20 °C). Lysate from sonicated cells (Supporting Information) was used for purification. Except *Bc*GT1 that was purified by anion exchange chromatography, all enzymes were purified by affinity chromatography *via* their His- or Strep-tag. The imidazole used for elution of His-tagged enzymes was carefully removed by threefold buffer exchange in ultrafiltration concentrator tubes. The methods used for enzyme purification are summarized in the Supporting Information, and enzyme purity was documented by SDS PAGE (Supporting Information Figure S1). Enzymes were stored in suitable buffers (Supporting Information; UGT1A9, 10 mg/mL; UGT71E5, arbutin synthase, 15–25 mg/mL; *Bc*GT1, UGT71A15, UGT708A6, 30–50 mg/mL; OleD wildtype, 50–70 mg/mL; and OleD triple mutant ASP, 300 mg/mL) and at −80 °C. Preparations were stable for at least 4–8 weeks. Before use, enzymes were checked for specific activity. A DeNovix DS-11+ spectrophotometer (DeNovix Inc., Wilmington, DE, USA) was used for protein determination. Molecular weight and molar extinction coefficients were calculated using the ProParam tool in ExPASy.

### Enzyme Activity Assay

Activity for glycosylation of the standard acceptor substrate from UDP-glucose was determined as described in the literature.^32−34,37−39,46^ The assay conditions used (acceptor substrate, buffer, and temperature) are summarized in Table 1. Reactions were done in 0.3 mL total volume and 0.1–5.0 mg/mL enzyme was employed. Incubation was done in a Thermomixer Comfort instrument (Eppendorf, Hamburg, Germany) with agitation rate at 400 rpm. Samples (20–30 μL) were taken at certain times (up to 22 h), and reaction was quenched with the same volume of ice-cold acetonitrile. Consumption of the acceptor substrate (1.0 mM) in the supernatant was measured by HPLC. One unit of activity is the enzyme amount consuming 1 μmol acceptor/min under the specified conditions.

**Table 1:** Panel of Leloir GTs for Glycosylation of 15-Hydroxy Cinmethylin^a^^,^^b^

enzyme (origin)	acceptor substrate/specific activity^c^	glycosylation of 15-hydroxy cinmethylin^b^		
		mono-glycosylation activity	15-hydroxy cinmethylin conversion^d^^,^^e^	disaccharide glycoside^f^
*Bc*GT1 (*B. cereus*)	4-methyl umbelliferone/0.21 mU mg^–1^	60 mU mg^–1^	65%^d^	11%
OleD wildtype (*S. antibioticus*)	4-methyl umbelliferone/1.0 mU mg^–1^	2.5 mU mg^–1^	82%^e^	13%
OleD triple variant, ASP (*S. antibioticus*)	4-methyl umbelliferone/2.0 mU mg^–1^	2.9 mU mg^–1^	86%^e^	14%
UGT71E5 (*C. tinctorius*)	7-amino-4-methylcoumarin/24.9 mU mg^–1^	279 mU mg^–1^ (pH 7.4), 431 mU mg^–1^ (pH 9.0)	98%^d^	N.D.
Arbutin synthase (*R. serpentina*)	phloretin/0.026 mU mg^–1^	N.D.	N.D.^e^	N.D.
UGT71A15 (*M. domestica*)	phloretin/2.4 mU mg^–1^	1.4 mU mg^–1^	40%^d^	N.D.
UGT708A6 (*Z. mays*)	phloretin/0.9 mU mg^–1^	N.D.	N.D.^e^	N.D.
UGT1A9 (Homosapiens)	4-methyl umbelliferone/0.85 mU mg^–1^	N.D.	N.D.^e^	N.D.

a N. D. Not detectable.

b Specific activities are from triplicate determinations (*N* = 3) and have standard errors of 35% or less of the reported mean value. Conversion data and product distributions are from a single experiment, confirmed in one biological replicate (*N* = 2).

c The following conditions (taken from the literature on the respective enzyme) were used for measurement of specific activity and for glycosylation of 15-hydroxy cinmethylin. *Bc*GT1: 0.5 mg/mL, 37 °C, 100 mM HEPES (pH 7.4), 5 mM MgCl_2_; OleD wildtype and triple mutant ASP: 2.0 mg/mL, 25 °C, 50 mM Tris–HCl (pH 8.0), 5 mM MgCl_2_; UGT71E5: 0.1 mg/mL, 30 °C, 50 mM Tris–HCl (pH 7.4, pH 9.0), 5 mM MgCl_2_; arbutin synthase: 5.0 mg/mL, 37 °C, 100 mM Tris–HCl (pH 7.5), 10 mM MgCl_2_; UGT71A15: 3.0 mg/mL, 30 °C, 50 mM HEPES (pH 8.0), 50 mM KCl, 13 mM MgCl_2_; UGT708A6: 4.1 mg/mL, 30 °C, 50 mM HEPES (pH 8.0), 50 mM KCl, 13 mM MgCl_2_; UGT1A9: 2.5 mg/mL, 37 °C, 50 mM Tris–HCl (pH 7.4), 3.5 mM MgCl_2_.

d Percent conversion of 15-hydroxy cinmethylin (1.0 mM) after 6 h of incubation.

e Percent conversion of 15-hydroxy cinmethylin (1.0 mM) after 23 h of incubation.

f Percent disaccharide glycoside in the total product formed from 15-hydroxy cinmethylin after 23 h of incubation.

### Glycosylation of 15-Hydroxy Cinmethylin

Reactions were performed at 0.3 mL total volume in Eppendorf tubes, using agitation at 400 rpm with the Thermomixer Comfort. The conditions used (buffer, temperature, and enzyme concentration) varied slightly among the different enzymes and are detailed in Table 1. The 15-hydroxy cinmethylin was used at 1.0 mM [4% dimethyl sulfoxide (DMSO), by volume] in the presence of twofold excess of UDP-glucose and UDP-glucuronic acid (used only for UGT1A9). The reaction was started by adding the enzyme (pre-incubated at reaction temperature for 2 min) to the substrate solution. To stop the reaction, ice-cold acetonitrile was added to the sample (1:1, by volume), and incubation was done on ice for 10 min. The precipitated enzyme was filtered off, and the liquid was analyzed further. Samples were taken at suitable times to measure the initial reaction rates (≤1 h) and to determine the course of conversion (up to 24 h).

### Reversed-Phase HPLC Analytics

Samples were analyzed on an Agilent 1200 HPLC system (Santa Clara, CA, USA) equipped with a Kinetex EVO C18 column (5 μm, 100 Å, 150 × 4.6 mm; Phenomenex, Aschaffenburg, Germany) and a UV detector. The column temperature was 45 °C. The injection volume was 5–10 μL. The eluent flow rate was 1 mL/min. The column was equilibrated in water containing 0.1% formic acid. Elution was done with an increasing gradient of acetonitrile (0.1% formic acid), starting from 10%.

#### Assays

Reactions with 4-methylumbelliferone and 7-amino-4-methylcoumarin were analyzed with 10%–60% acetonitrile over 15 min. The column was washed with 90% acetonitrile for 2 min and equilibrated with 10% acetonitrile for 3 min. The acceptors and the corresponding β-d-glucosides were detected at 220 and 320 nm. Reactions with phloretin were analyzed as described in the literature.^32,33^ Detection of the acceptor and the corresponding β-d-glucoside was at 288 nm.

#### Reactions with 15-Hydroxy Cinmethylin

A gradient of 20–75% acetonitrile over 5.5 min was used. The column was washed with 75% acetonitrile for 2 min and equilibrated with 20% acetonitrile for 4.5 min. 15-Hydroxy cinmethylin and its mono-β-d-glucoside and di-β-d-glucosides were detected at 203 nm.

### NMR

The β-d-glucoside isolated from the reaction with 15-hydroxy cinmethylin (10 mM) was analyzed. Briefly, the product was purified by reversed-phase HPLC using the Kinetex EVO C18 column used also for analytical determinations. Pooled fractions (15 mL) were concentrated to about one-twentieth of the original volume using a Heidolph Laborota 4000 rotary evaporator equipped with a Vacuubrand PC2001 pump and CVC2000II controller (Wertheim, Germany; 40 °C, <230 mbar) and then lyophilized overnight with an Alpha 1–4 freeze dryer (Martin Christ Gefriertrocknungsanlagen GmbH, Osterode am Harz, Germany) at −40 °C and 0.020 mbar. The product was taken up in DMSO-*d*_6_ (99.8% D) for NMR measurements. A Varian Unity Inova 500 MHz spectrometer (Agilent Technologies) and VNMRJ 2.2D software were used. ^1^H- and ^13^C NMR, COSY, HSQC, and HMBC spectra were recorded. The spectra of the enzymatically synthesized product enabled unambiguous assignment of its structure as 15-hydroxy cinmethylin β-d-glucoside. The spectra are shown in the Supporting Information Figures S2–S4, and the spectral data are summarized in the Supporting Information Table S1.

### LC–MS

A Shimadzu LCMS-2020 system (Kyoto, Japan) equipped with a Nucleodur C18 gravity column (3 μm, 110 Å, 150 × 3 mm, Macherey-Nagel, Düren, Germany) was used. A linear gradient (10 to 85%) of acetonitrile in ammonium acetate buffer (5 mM, pH 6.67) over 5 min was used. The column was washed with 10% acetonitrile for 2.5 min. The flow rate was 0.7 mL/min. The column temperature was 30 °C. A UV detector tuned to 210 and 262 nm was used. Masses were scanned over the range of 150–800 in the positive mode. The masses of mono-glucoside (452.5; [M + H]^+^, 453.5; [M + Na]^+^, 475.5; and [M + K]^+^, 491.5) and bis-glucoside (614; [M + H]^+^, 615; [M + Na]^+^, 637.6; and [M + K]^+^, 653.6) were also analyzed in the SIM mode. The obtained data are shown in the Supporting Information Figure S5.

## Results and Discussion

### Panel of Leloir GTs for 15-Hydroxy Cinmethylin Glycosylation

To identify enzyme(s) for β-glycosylation of 15-hydroxy cinmethylin, we selected a representative panel of eight GTs (Table 1) active with UDP-glucose and showing broad specificity for bulky acceptor substrates. We chose a balanced distribution between GTs of plant (four enzymes) and microbial origin (three enzymes). Among the bacterial GTs, the OleD from *S. antibioticus* is a well-characterized enzyme that has been widely used for small-molecule glycosylation.^38,39^ Its triple variant was laboratory-evolved for even broadened donor and acceptor scope.^38,39^ OleD wildtype and its triple mutant ASP are active with primary alcohols and benzyl alcohols as in 15-hydroxy cinmethylin in particular.^38,39^ We additionally used the human GT UGT1A9 to examine β-glucuronidation of 15-hydroxy cinmethylin from UDP-glucuronic acid. Using recombinant production in *E. coli*, we obtained the GTs in a highly purified form (Supporting Information Figure S1). Reaction with the standard acceptor substrate from the literature revealed that each enzyme was functional, showing the required activity for glycosylation from UDP-glucose (Table 1) and suitable for test of reactivity with 15-hydroxy cinmethylin.

**Figure 2:**
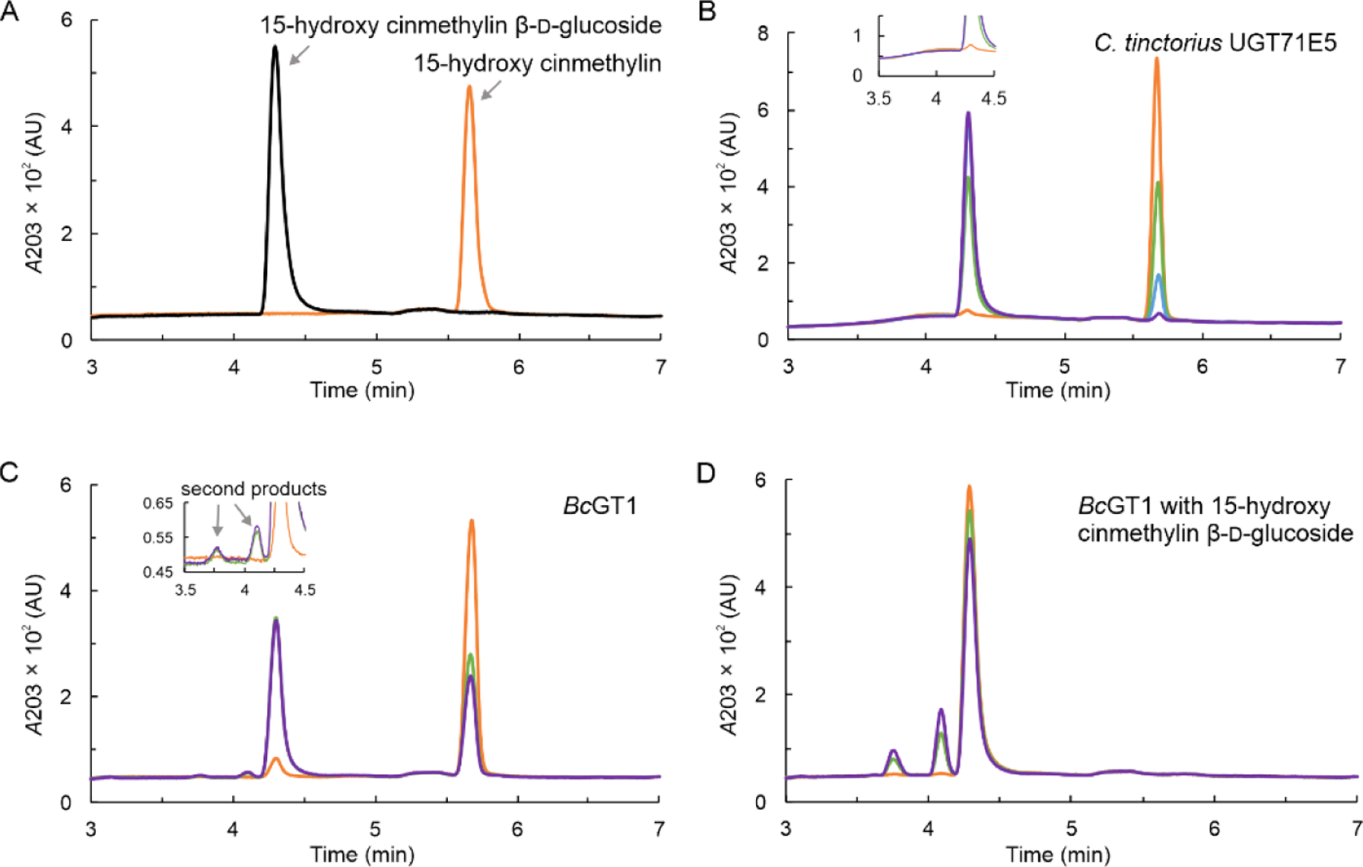
HPLC analysis of enzymatic glycosylation of 15-hydroxy cinmethylin. (A) 15-hydroxy cinmethylin (acceptor, orange) and 15-hydroxy cinmethylin β-d-glucoside (product, black). (B,C) Samples of reactions of *C. tinctorius* UGT71E5 (B; pH 7.4; 0 h, orange; 0.5 h, green; 2 h, blue; 6 h, purple) and *Bc*GT1 (C; pH 7.4; 0 h, orange; 1 h, green; 6 h, purple) with 15-hydroxy cinmethylin (1 mM) and UDP-glucose (2 mM). The insets show close-ups of the HPLC traces to highlight additional products (peaks at 3.7 and 4.1 min) formed by *Bc*GT1 but not by UGT71E5. (D) Glycosylation products (HPLC traces at 0 h, orange; 0.5 h, green; and 3 h, purple) formed upon reaction of *Bc*GT1 with 15-hydroxy cinmethylin β-d-glucoside (1 mM) and UDP-glucose (2 mM). From their elution, the products formed are the same as the additional glycosylation products from the reaction of 15-hydroxy cinmethylin (panel C). Disaccharide-bearing products of the general form 15-hydroxy cinmethylin β-d-glucosyl-β-d-glucoside are suggested.

HPLC trace of the sample from the UGT71E5-catalyzed conversion of 15-hydroxy cinmethylin in the presence of UDP-glucose revealed the appearance of a new compound peak (Figure 2) that increased in abundance as the 15-hydroxy cinmethylin consumption progressed. The mass data (452.5; [M + H]^+^, 453.5; [M + Na]^+^, 475.5; and [M + K]^+^, 491.5) for the product are fully consistent with those of singly glycosylated 15-hydroxy cinmethylin. As shown later, we isolated the compound peak and showed with NMR that it corresponded to the expected 15-hydroxy cinmethylin β-d-glucoside (Figure 1). With HPLC analytics properly referenced and calibrated, we performed screening of the GT panel. We found that above a detection limit of 0.1% 15-hydroxy cinmethylin conversion within 24 h, arbutin synthase and UGT708A6 were inactive. UGT1A9 was also inactive, irrespective of whether UDP-glucuronic acid or UDP-glucose was used as the donor substrate. The *Bc*GT1, OleD in the wildtype and triple variant form, UGT71A15, and UGT71E5 were active (Figure 2 and Table 1).

Initial rates of 15-hydroxy cinmethylin glycosylation were determined, and specific activities calculated from the data are summarized in Table 1. UGT71E5 was the most active among the GTs tested. *Bc*GT1 was 4.5-fold less active. With a specific activity below ∼5 mU/mg, the OleD enzymes and the UGT71A15 were usable for the characterization of the 15-hydroxy cinmethylin glycosylation, but these enzymes were not considered for preparative synthesis. The glycosylation of an acceptor alcohol from UDP-glucose is pH-dependent in the pH range ≥ ∼6.5 where the released UDP is fully deprotonated (eq 1).^48^ In terms of reaction equilibrium, glycosylation is thus favored at high pH. The UGT71E5 showed higher specific activity at pH 9.0 than at pH 7.4 (Table 1), thus rendering the enzyme a promising candidate for the synthesis of 15-hydroxy cinmethylin β-d-glucoside (Figure 1) at high pH.
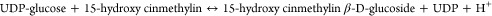
1

### Time Course Analysis of the 15-Hydroxy Cinmethylin Glycosylation from UDP-Glucose

**Figure 3:**
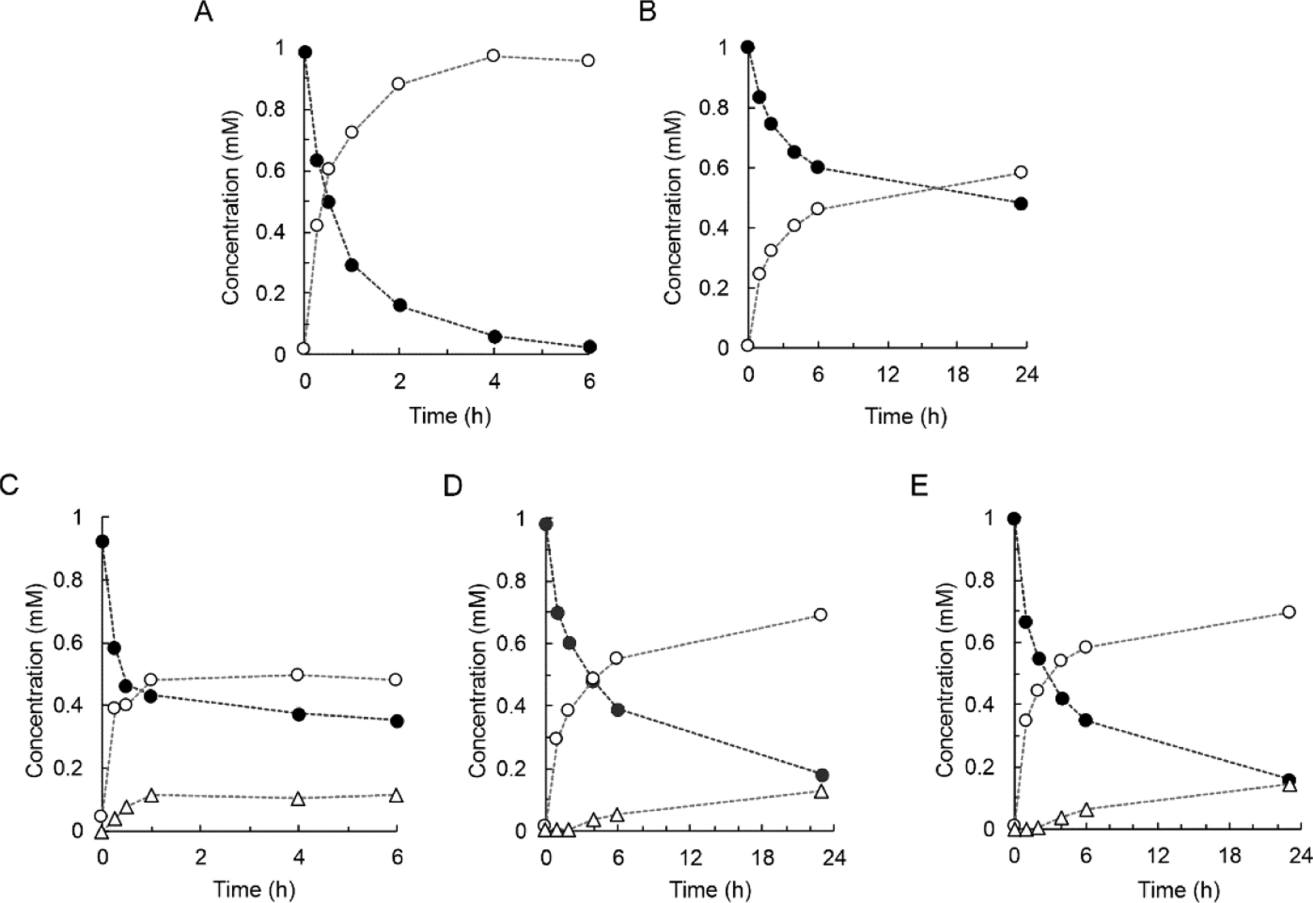
Time courses of enzymatic glycosylation of 15-hydroxy cinmethylin. Reactions used 2 mM UDP-glucose. The 15-hydroxy cinmethylin β-d-glucoside (open circles), the 15-hydroxy cinmethylin (closed circles), and the putative disaccharide glycoside of 15-hydroxy cinmethylin (open triangles) are shown. (A) UGT71E5; (B) UGT71A15; (C) *Bc*GT1; (D) OleD wildtype; and (E) OleD triple variant ASP. The concentration of the putative disaccharide glycosides of 15-hydroxy cinmethylin was obtained as the sum of the two product peaks at 3.7 and 4.1 min, as shown in Figure 2C. Initial rates of 15-hydroxy cinmethylin β-d-glucoside formation were calculated from the data and are shown in Table 1.

Conversion of 15-hydroxy cinmethylin was analyzed for each GT, and the corresponding reaction time courses are shown in Figure 3 (panels A–E). UGT71E5 promoted a “clean” transformation (Figure 3A) that gave the desired mono-β-d-glucoside (Figure 1) as a single product in excellent yield (≥95%) within just 6 h at a comparably low enzyme loading (0.1 mg/mL). Despite 30-times higher enzyme loading being used, reaction of the UGT71A15 (Figure 3B) proceeded in lower yield (∼60%) within 24 h. It was selective in that only 15-hydroxy cinmethylin β-d-glucoside was formed.

**Figure 4:**
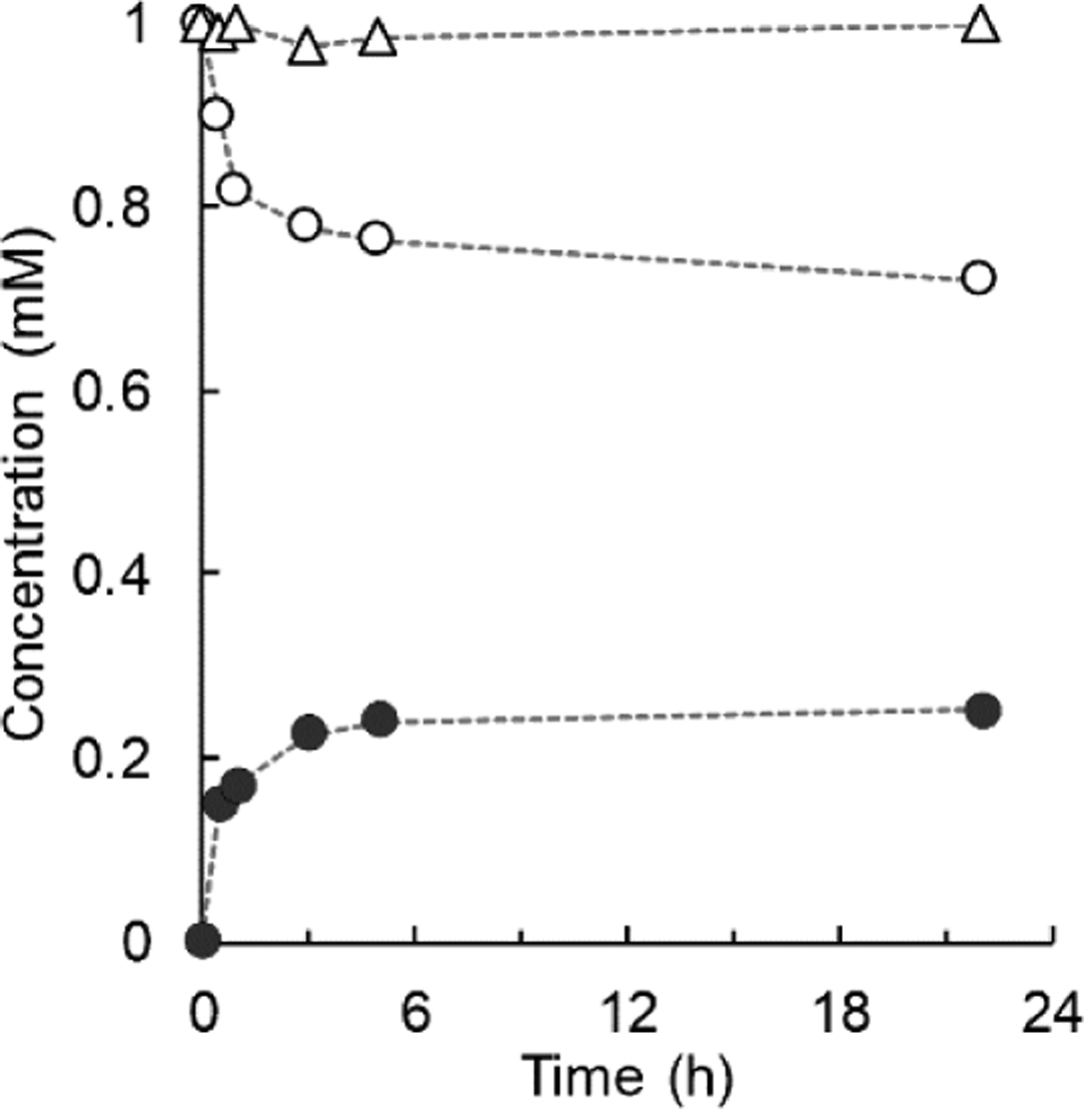
Glycosylation of 15-hydroxy cinmethylin β-d-glucoside by *Bc*GT1. The reaction used 2 mM UDP-glucose and 0.5 mg/mL *Bc*GT1. The symbols show 15-hydroxy cinmethylin β-d-glucoside (open circles, 1 mM) and the putative disaccharide glycosides of 15-hydroxy cinmethylin (closed circles). The concentration of the disaccharide-modified 15-hydroxy cinmethylin was obtained as the sum of the two product peaks at 3.7 and 4.1 min, as shown in Figure 2C. The control lacking *Bc*GT1 is shown in open triangles.

Using *Bc*GT1 (0.5 mg/mL), we observed fast reaction for ∼65% conversion of 15-hydroxy cinmethylin. Important difference to the product-selective reactions of UGT71E5 and UGT71A15 was that *Bc*GT1 released additional products (∼11%; Table 1), detectable as two new peaks eluting earlier than the target product (15-hydroxy cinmethylin β-d-glucoside) in the HPLC trace of the sample from the reaction (Figure 2C). The elution characteristics were consistent with the new products exhibiting higher polarity than the 15-hydroxy cinmethylin β-d-glucoside. From their mass data ([M + Na]^+^, 637.6; [M + K]^+^, 653.6; Supporting Information Figure S5), the products had arisen from an iterative, double glycosylation of the 15-hydroxy cinmethylin. Since 15-hydroxy cinmethylin only has a single site for glycosylation (the C15 hydroxy group, Figure 1), the products formed must be disaccharide glycosides derived according to the glycosylation sequence, 15-hydroxy cinmethylin → 15-hydroxy cinmethylin β-d-glucoside → 15-hydroxy cinmethylin β-d-glucosyl β-d-glucoside. The suggestion for an iterative glycosylation of 15-hydroxy cinmethylin was consistent with the product formation kinetics in the *Bc*GT1 reaction (Figure 3C): the doubly glycosylated products only appeared in the mixture after the mono-glycoside had been released in substantial amounts. Moreover, we showed that purified 15-hydroxy cinmethylin β-d-glucoside (Figure 1) was the substrate for further glycosylation from UDP-glucose catalyzed by the *Bc*GT1 (Figures 2D and 4). Reaction with 15-hydroxy cinmethylin β-d-glucoside gave the same disaccharide glycoside products as identified from reaction with 15-hydroxy cinmethylin (Figure 2D). The rate of glycosylation of 15-hydroxy cinmethylin β-d-glucoside determined from Figure 4 (6.5 mU/mg) was ∼9.2-fold lower than the glycosylation rate of 15-hydroxy cinmethylin. Interestingly, *Bc*GT1 reaction with 15-hydroxy cinmethylin stopped after ∼1 h (Figure 3C), despite the fact that a substantial portion of the acceptor substrate (∼35%) was still remaining. We noted that the UDP-glucose was largely depleted at this point, implying that the substrate had been used in ways (*e.g.*, hydrolysis of UDP-glucose) not completely accounted for by our analytical procedures. Considering the focus of this study on the synthesis of 15-hydroxy cinmethylin β-d-glucoside, we did not pursue these characteristics of the *Bc*GT1 reaction, leaving them for future study. Reactions of the OleD enzymes (Figure 3D,E) involved iterative glycosylation of the 15-hydroxy cinmethylin similarly as with *Bc*GT1. The conversion of 15-hydroxy cinmethylin was ∼86%, higher than in the *Bc*GT1 reaction. Iterative glycosylation of small-molecule acceptors was previously reported for both *Bc*GT1 and OleD. The flavonoid kaempferol was converted into the di- or tri-*O*-β-d-glucoside by *Bc*GT1.^49^ Glycosylation of thiophenol by OleD gave a disaccharide glycoside product, namely, the 2′-*O*-(β-d-glucosyl)-β-d-thioglucoside.^39^

Based on requirements of reaction selectivity, we chose UGT71E5 and UGT71A15 for biocatalytic synthesis of the 15-hydroxy cinmethylin β-d-glucoside.

### Preparative Synthesis of 15-Hydroxy Cinmethylin β-d-Glucoside

The UGT71A15 showed low activity for glycosylation of 15-hydroxy cinmethylin (Table 1), and the yield of 15-hydroxy cinmethylin β-d-glucoside did not exceed ∼60% (∼0.6 mM; Figure 3B). To examine limitations on UGT71A15 synthetic utility caused by the reaction conditions, we conducted the synthesis in the presence of an enzyme stabilizer [tris(2-carboxyethyl)phosphine; up to 5.0 mM] and used varied concentrations (1.0–5.0 mM) of UDP-glucose. We also applied *in situ* formation of UDP-glucose *via* the sucrose synthase reaction (Figure 1B). The results are shown in the Supporting Information Figures S6–S9. The formation of 15-hydroxy cinmethylin β-d-glucoside was marginally improved by these changes in reaction conditions. We thus concluded that UGT71A15 was not a likely candidate enzyme for successful application in the synthesis of 15-hydroxy cinmethylin β-d-glucoside.

**Figure 5:**
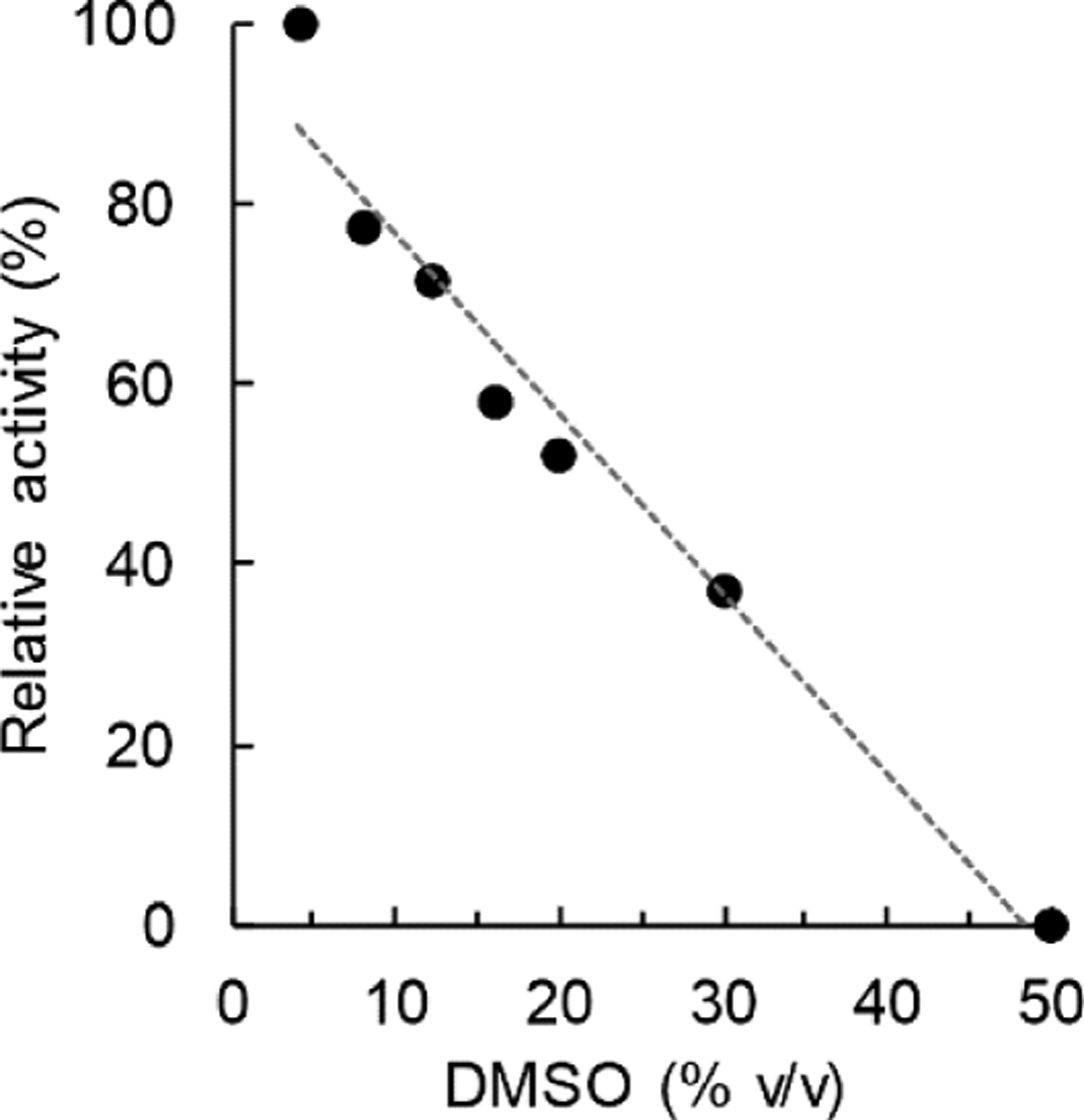
Effect of the DMSO co-solvent on the activity of UGT71E5 for glycosylation of 15-hydroxy cinmethylin. Assays were performed at pH 9.0 using 1 mM 15-hydroxy cinmethylin and 2 mM UDP-glucose. UGT71E5 was used at 0.1 mg/mL. The reaction time was 6 h.

**Figure 6:**
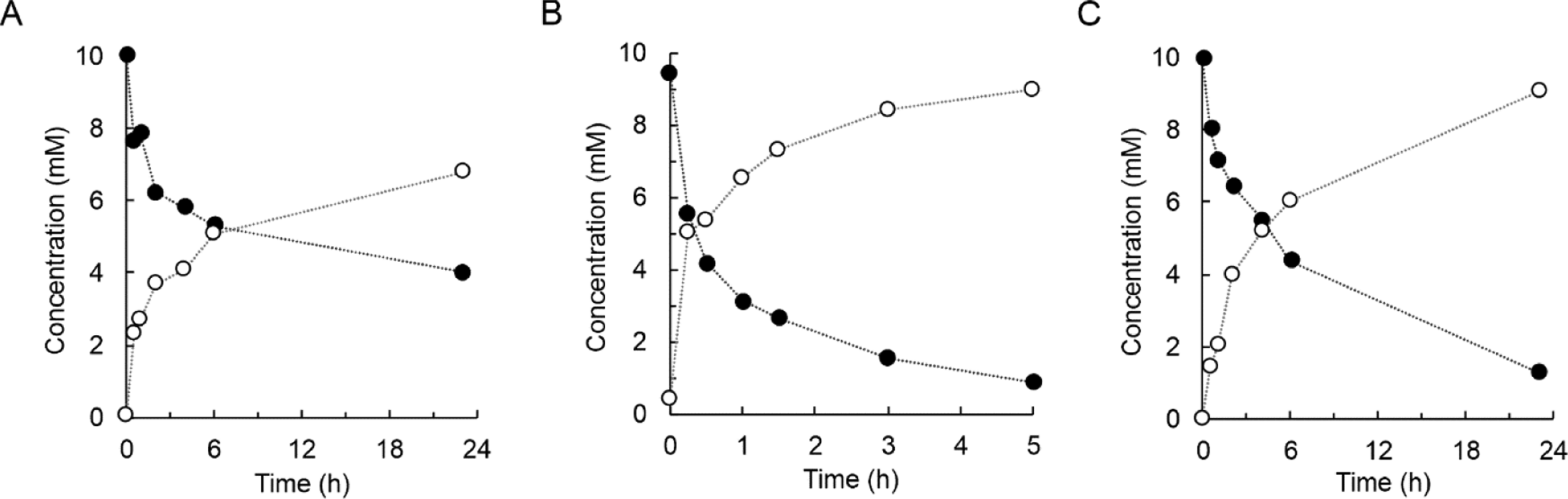
Preparative synthesis of 15-hydroxy cinmethylin β-d-glucoside by UGT71E5. 15-Hydroxy cinmethylin (10 mM; closed circles) and 15-hydroxy cinmethylin β-d-glucoside (open circles) are shown. UGT71E5 was used at 0.5 mg/mL. The DMSO concentration was 10% (by volume). The reaction volume was 0.3 mL. (A,B) Sodium phosphate [(A); 50 mM, pH 7.0] and Tris buffer [(B), 50 mM, pH 9.0] additionally containing 5 mM MgCl_2_ were used. The UDP-glucose concentration was 10 mM (A) and 15 mM (B). (C) Reaction involving UDP-glucose regeneration from sucrose (100 mM) and UDP (1.0 mM) by *Gm*Susy (0.1 mg/mL, 4.1 U/mg) at pH 7.0.

Having selected UGT71E5, we analyzed the effect of the DMSO co-solvent on the enzyme activity. The co-solvent was necessary to enhance the 15-hydroxy cinmethylin solubility to a minimum target concentration of 10 mM. UGT71E5 activity was strongly inhibited by DMSO (Figure 5), with half of the original activity lost at 25% co-solvent (by volume). DMSO at 10% was a useful compromise between 15-hydroxy cinmethylin solubility enhancement to ∼10 mM and retention of UGT71E5 activity (∼80%). The time course of 15-hydroxy cinmethylin conversion at pH 7.0 is shown in Figure 6A. After 23 h, the yield of 15-hydroxy cinmethylin β-d-glucoside was ∼68%. The product selectivity was retained. Bis-glycoside formation was below the detection limit (≤0.05 mM). Considering the pH effect on enzyme activity (Table 1), we performed the synthesis at pH 9.0 (Figure 6B). The enzymatic reaction rate was enhanced ∼6-fold (360 mU/mg), and the yield was increased to ∼90%. To avoid use of UDP-glucose in concentrations equaling the 15-hydroxy cinmethylin concentration, we also performed the reaction under UDP-glucose recycling (Figure 6C) from sucrose. Sucrose synthase (0.1 mg/mL; 4.1 U/mg) was applied to make the 15-hydroxy cinmethylin glycosylation rate-limiting overall. Earlier studies of the kinetics and thermodynamics of similar GT cascade reactions suggested that the UDP-glucose recycling was best conducted at a pH of ∼7.0.^42,43,50^ The 15-hydroxy cinmethylin β-d-glucoside was obtained in 91% yield after 23 h. Its synthesis involved nine times the use of the UDP/UDP-glucose shuttle. The final product concentration was 4.1 g/L in 0.3 mL. The conversion rate was consistent with that of UGT71E5 reaction at pH 7.0 using UDP-glucose (Figure 6A). A specific UGT71E5 activity of ∼61 and ∼66 mU/mg was calculated from the time courses in Figure 6A,C, respectively.

The 15-hydroxy cinmethylin β-d-glucoside was isolated in high purity (isolated yield: ≥95%, ∼1.1 mg) by preparative HPLC. The product was characterized by one- and two-dimensional ^1^H and ^13^C NMR methods. Results are shown in the Supporting Information Figures S2–S4 and Table S1. The expected β-d-glucoside product structure (Figure 1) was confirmed unambiguously. Hydrogen peaks of the β-d-glucosyl residue bound to 15-hydroxy cinmethylin are shown in 2.9–5.1 ppm in ^1^H NMR and HSQC spectra (Supporting Information Figures S2 and S3). Mass data (452.5; [M + H]^+^, 453.5; [M + Na]^+^, 475.5; and [M + K]^+^, 491.5) are consistent with the product structure. The product is a ∼1:1 mixture of diastereomers due to β-glucosylated isomers of the 15-hydroxy cinmethylin (Supporting Information Figure S2, panel B). In glycosylating 15-hydroxy cinmethylin from UDP-glucose, UGT71E5 appears to be not selective regarding the 15-hydroxy cinmethylin isomers (Figure 1).

In summary, therefore, the herein-performed screening of permissive Leloir GTs discovers UGT71E5 for its useful activity to glycosylate 15-hydroxy cinmethylin from UDP-glucose and demonstrates efficient biocatalytic synthesis of the desired β-d-glucoside (15-hydroxy cinmethylin β-d-glucoside). The target product is obtained in a yield of ≥90% based on the 15-hydroxy cinmethylin supplied. Using a co-solvent to the extent tolerated by UGT71E5 to increase solubility of the 15-hydroxy cinmethylin acceptor, the final concentration of 15-hydroxy cinmethylin β-d-glucoside reaches 4.1 g/L (in 0.3 mL). UDP-glucose recycling from sucrose and UDP enables a high yield of 15-hydroxy cinmethylin β-d-glucoside in a reaction mixture conducive to facile product recovery. The synthetic 15-hydroxy cinmethylin β-d-glucoside supports biological studies of cinmethylin metabolism in the environment.

## Supporting Information Available

Enzyme expression and purification procedures; SDS-polyacrylamide gel showing the purified GTs; NMR spectra of 15-hydroxy cinmethylin β-d-glucoside; mass analysis of the putative disaccharide glycosides of 15-hydroxy cinmethylin produced by *Bc*GT1; effects of TCEP, enzyme concentration, and UDP-glucose concentration on 15-hydroxy cinmethylin glycosylation by UGT71A5; and two-enzyme cascade glycosylation of 15-hydroxy cinmethylin with UGT71A15 and sucrose synthase (PDF)

## Abbreviations

UDP: uridine 5′-diphosphate
GT: glycosyltransferase
TCEP: tris(2-carboxyethyl)phosphine
*Gm*Susy: sucrose synthase from soybean *Glycine max*
