# Supplementary material for: Selective β-Mono-Glycosylation of a C15-Hydroxylated Metabolite of the Agricultural Herbicide Cinmethylin Using Leloir Glycosyltransferases
Source: J Agric Food Chem. 2021 May 11;69(19):5491–9. doi: 10.1021/acs.jafc.1c01321 (PMC8278484; doi:10.1021/acs.jafc.1c01321)
Supplement: Supplementary file 1 — jf1c01321_si_001.pdf [file jf1c01321_si_001.pdf]

Selective β-Mono-Glycosylation of a C15-Hydroxylated Metabolite of the Agricultural Herbicide Cinmethylin Using Leloir Glycosyltransferases

Jihye Jung[a], Katharina Schmölzer[a], Doreen Schachtschabel[b], Michael Speitling[b], and Bernd Nidetzky[a,c,*]

[a] Austrian Centre of Industrial Biotechnology, A-8010 Graz, Austria

[b] BASF SE, Carl-Bosch-Strasse 38, 67056 Ludwigschafen, Germany

[c] Institute of Biotechnology and Biochemical Engineering, NAWI Graz, TU Graz, A-8010 Graz, Austria

[*] Corresponding author. Phone: +43-316-873-8400; Email: bernd.nidetzky@tugraz.at

Supporting Information

Table of contents

Enzyme expression and purification

Supporting Table S1. 1D- and 2D-NMR spectral data of 15-hydroxy cinmethylin β-D-glucoside extracted from Supporting Figure S2-S4.

Supporting Figure S1. SDS polyacrylamide gel showing the purified glycosyltransferases.

Supporting Figure S2. 1D-NMR spectra of 15-hydroxy cinmethylin β-D-glucoside produced by *C. tinctorius* UGT71E5.

Supporting Figure S3. 2D-HSQC spectrum of 15-hydroxy cinmethylin β-D-glucoside produced by *C. tinctorius* UGT71E5.

Supporting Figure S4. 2D-HMBC and COSY spectra of 15-hydroxy cinmethylin β-D-glucoside produced by *C. tinctorius* UGT71E5.

Supporting Figure S5. Mass analysis of 15-hydroxy cinmethylin β-D-glucoside synthesized enzymatically and bis-glycosides formed by *Bc*GT1.

Supporting Figure S6. Effect of TCEP concentration on glycosylation of 15-hydroxy cinmethylin by UGT71A15.

Supporting Figure S7. Effect of enzyme amounts on glycosylation of 15-hydroxy cinmethylin with UGT71A15.

Supporting Figure S8. Effect of UDP-glucose concentration on the formation of 15-hydroxy cinmethylin β-D-glucoside by UGT71A15.

Supporting Figure S9. Two-enzyme cascade glycosylation of 15-hydroxy cinmethylin by UGT71A15 and *Gm*Susy.

References

Enzyme expression and purification

Escherichia coli BL21 (DE3) was used as expression strains for *Bc*GT1[1] and UGT1A9[2]. *Bc*GT1 (expression plasmid: pET 15b) and UGT1A9 (expression plasmid : pMALc5xHis) were produced as follows. Cells were cultivated in 1 L baffled shake flasks containing 250 mL LB medium supplemented with 115 µg mL^{-1} ampicillin at 37 °C and 110 rpm. When an optical density at 600 nm (OD_{600}) reaches between 0.9-1.0, isopropyl β-D-1-thiogalactopyranoside (IPTG; 1 mM for *Bc*GT1; 0.5 mM for UGT1A9) was added and then the cells were incubated at 18 °C for 20h. Cells were harvested by Sorvall RC-5B Superspeed Centrifuge (DuPont, Wilmington, DE, USA) at 4°C and 5000 rpm for 25 min. Collected cells were resuspended in sodium phosphate buffer (25 mM, pH 7.0) containing 5 mM dithiothreitol for *Bc*GT1 and in sodium phosphate buffer (30 mM, pH 7.4) containing 150 mM NaCl for UGT1A9. Cells were disrupted by Fisherbrand™ Model 505 Sonic Dismembrator (Waltham, MA, USA; 10 s on, 20s off, amplitude 30%, 6 min total on-time). Cell-free extract solution was obtained by centrifugation at 5000 rpm and 4°C for 60 min and was filtered through a 1.2 µm filter before purification. *Bc*GT1 was purified by ion-exchange chromatography with HiTrap DEAE FF column (5 mL, Cytiva, Chicago, IL, USA). *Bc*GT1 was eluted with 10% of sodium phosphate buffer (25 mM, pH 7.0) containing 5 mM dithiothreitol and 1 M NaCl. The eluted *Bc*GT1 solution was filtered off in triplicate at 4 °C and 5000 rpm (Vivaspin Turbo 15 PES, 10 kDa molecular weight cut-off, Sartorius, Göttingen, Germany). The corresponding buffer for cell resuspension was used. UGT1A9 was purified by using His-Trap FF column (5 mL, Cytiva). The column was washed with sodium phosphate buffer (30 mM, pH 7.4) containing 150 mM NaCl and 15 mM imidazole. A gradient method of 0% to 100% sodium phosphate buffer (30 mM, pH 7.4) containing 150 mM NaCl and 300 mM imidazole was used. Imidazole remained in the purified enzyme solution was filtered off in triplicate. The corresponding buffer used for cell resuspension were used.

OleD wildtype and tripe mutant ASP (expression plasmid: pET 28a (+))[3], UGT71E5 (expression plasmid: pET 28a(+))[4], arbutin synthase (expression plasmid: pSTRP3)[5], UGT71A15 (expression plasmid: pSTRP3)[6], and UGT708A6 (expression plasmid: pSTRP3)[5] were over-expressed and purified

by affinity chromatography, as described previously. OleD wildtype and triple mutant ASP were expressed in *Escherichia coli* Lemo 21. The enzymes were purified with His-Trap FF column. The purified enzyme solutions were washed off with Tris-HCl buffer (50 mM, pH 8.0) in triplicate. UGT71E5 was expressed in *Escherichia coli* BL21 (DE3) and purified with His-Trap FF column. Purified enzyme solution was washed off with Tris-HCl buffer (50 mM, pH 7.4) containing 50 mM NaCl and 1 mM tris(2-carboxyethyl)phosphine in triplicate. Arbutin synthase, UGT71A15, and UGT708A6 were expressed in *Escherichia coli* BL21-Gold (DE3). The enzymes were purified by StrepTrap-HP column (5 mL, Cytiva). The enzyme solutions were washed off with HEPES buffer (25 mM, pH 7.5) in triplicate.

Supporting Table S1. 1D- and 2D-NMR spectral data of 15-hydroxy cinmethylin β-D-glucoside extracted from Supporting Figure S2-S4.

Atom number	C Shift [a]	H Shift	H Multiplicity	H to C HMBC	COSY	C to H HMBC
1	33.43	1.45	m	2, 3, 5, 6, 17	4, 17	2, 4, 5, 17
2	31.48	1.45	m	1, 3, 5, 6, 17	4, 17	1, 4, 17, 18
4	41.54	1.45	m	1, 2, 3, 5, 6, 17	1, 2, 5	1, 2, 18
		1.97	m	2, 3, 5, 6		
3, 6	88.10 [b]					1, 2, 4, 5, 18, 19, 20
5	82.77	3.61	m	1, 3, 6, 8	4, 29	4, 17, 18
8	66.94	4.45	m	5, 9, 10, 14	15	5, 14
		4.57	d (*J* = 12.8 Hz)	5, 9, 10, 14	5	
9	136.13 [b]					8, 12, 13, 14, 15
10	137.62 [b]					8, 11, 12, 13, 15
11	128.29	7.47	d (*J* = 6.9 Hz)	10, 12, 13, 15	12, 13, 14	12, 13, 15
12, 13	127.12	7.29	m	9, 10, 11	11, 14	11, 14
14	127.61	7.39	d (*J* = 6.9 Hz)	8, 9, 12, 13	11, 12, 13	8, 12, 13
15	67.14	4.62	d (*J* = 12.3 Hz)	9, 10, 11, 22	8	8, 11, 22, 32
		4.90	m	9, 10, 11, 22	8, 31	
17	16.44	1.35	s	1, 2, 5	1, 2, 4	1, 2, 4
18	32.07	1.97	m	2, 3, 4, 19, 20	19, 20	19, 20
19, 20	17.90	0.91	d (*J* = 6.9 Hz)	3, 18, 19, 20	4, 18	18, 19, 20
22	102.01	4.20	two d (*J* = 7.7 Hz)	15, 27	27	15, 27, 32
24	76.81	3.09	m	25	25	28, 29, 30, 31
25	70.07	3.09	m	27	24	24, 26, 28, 29, 31
26	76.61	3.14	m		27	27, 32
27	73.39	3.03	m	22, 26	22, 32	32
28		4.90	m	25	32	
29	61.08	3.48	m	5, 24	5,30	30
	61.08	3.71	m	5, 25	5, 30	
30		4.57	d (*J* = 12.8 Hz)	24, 29	5	
31		4.90	m	24, 25	8, 28	
32		5.07	two d (*J* = 5.1 Hz)	22, 26, 27	27, 28, 31	

[a, b] The data were obtained from ^{13}C-NMR (Supporting Figure S2) [b] and 2D-NMR spectra (Supporting Figure S3-4).

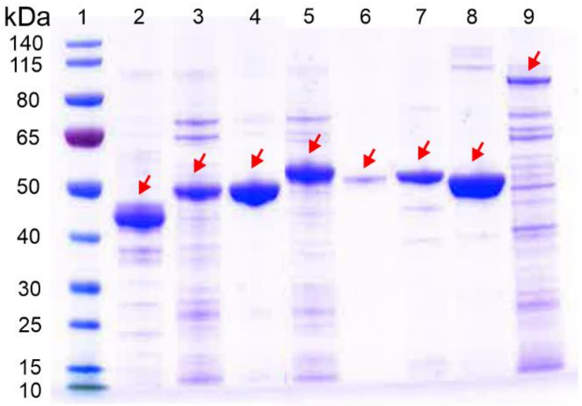

Supporting Figure S1. SDS polyacrylamide gel shows the purified glycosyltransferases. Protein amounts were each 5 µg. Lane 1: protein marker; Lane 2: *Bc*GT1 (45.5 kDa); Lane 3: OleD wildtype (47.3 kDa); Lane 4: OleD triple variant ASP (47.4 kDa); Lane 5: UGT71E5 (55.2 kDa); Lane 6: arbutin synthase (52.8 kDa); Lane 7: UGT71A15 (53.9 kDa); Lane 8: UGT708A6 (52.5 kDa); Lane 9: UGT1A9 (96.4 kDa).

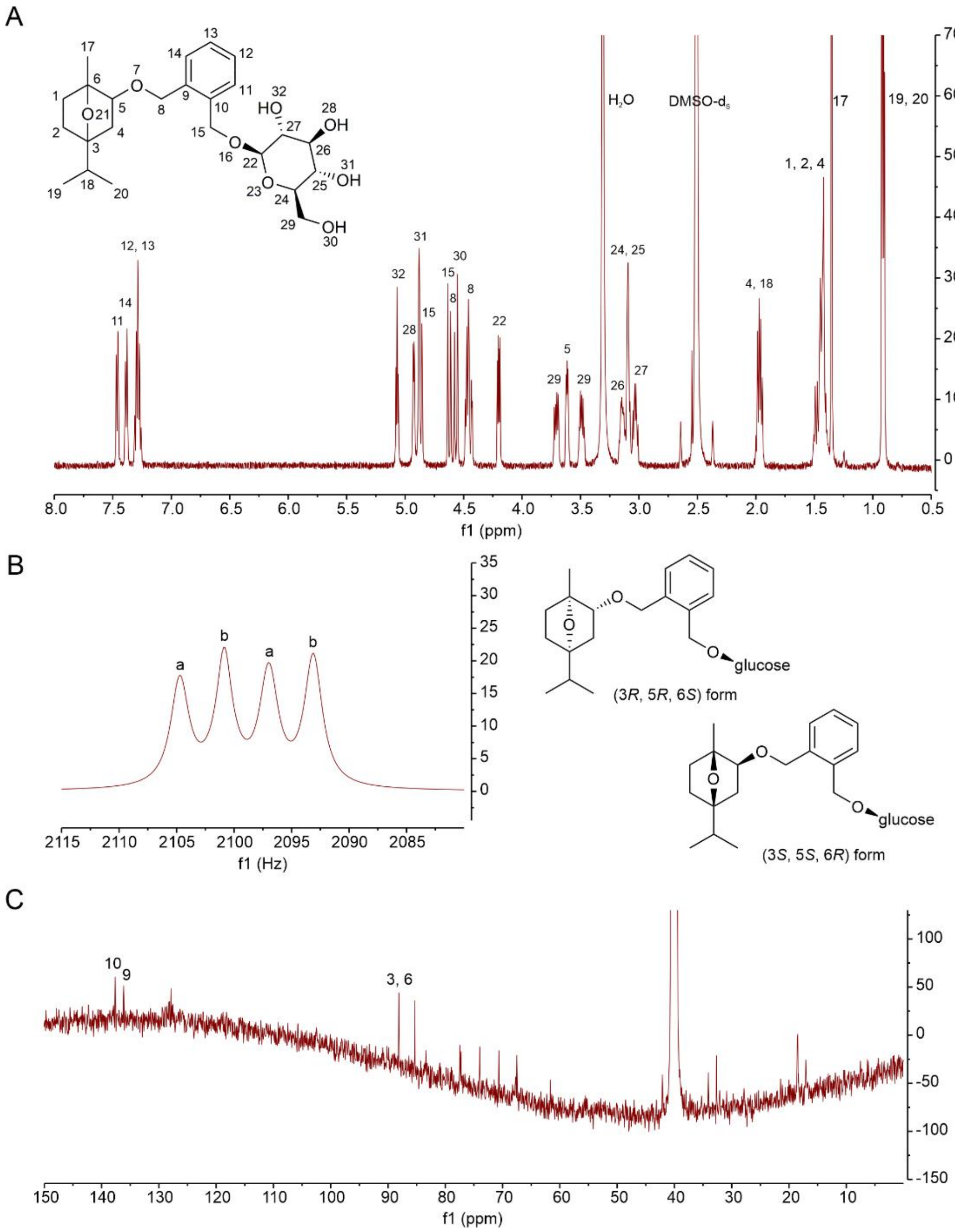

Supporting Figure S2. 1D-NMR spectra of 15-hydroxy cinmethylin β-D-glucoside produced by *C. tinctorius* UGT71E5. DMSO-d_6 was used as solvent. (A) ^{1}H-NMR. (B) Proton peaks of C22 of the diastereomeric 15-hydroxy cinmethylin β-D-glucoside in ^{1}H-NMR. Two doublets (a and b, coupling constant = 7.74 Hz) are shown with an area ratio of 9:10 (a, 0.45; b, 0.52). The diastereomeric structures are shown. (C) ^{13}C-NMR.

^{1}H NMR (500 MHz, DMSO-d_6) δ 0.91 (overlapping two d, 6 H, *J* = 6.9 Hz, 19-CH3, 20-CH3), 1.35 (s, 3 H, 17-CH3), 1.45 (m, 5 H, 1-CH2, 2-CH2, 4-CH2), 1.97 (m, 3 H, 4-CH2, 18-CH), 3.03 (m, 1 H, 27-CH), 3.09 (m, 2 H, 24-CH, 25-CH), 3.14 (m, 1 H, 26-CH), 3.48 (m, 1 H, 29-CH2), 3.61 (m, 1 H, 5-CH), 3.71 (m, 1 H, 29-CH2), 4.20 (overlapping two d from diastereomers, 1 H, *J* = 7.9 Hz, 22-CH), 4.45 (m, 2 H, 8-CH2, 30-OH), 4.57 (d, 1 H, *J* = 12.8 Hz, 8-CH2), 4.62 (d, 1 H, *J* = 12.3 Hz, 15-CH2), 4.90 (m, 3 H, 15-CH2, 31-OH, 28-OH), 5.07 (overlapping two d from diastereomers, 1 H, *J* = 5.1 Hz, 32-OH), 7.29 (m, 2 H, 12-CH, 13-CH), 7.39 (d, 1 H, *J* = 6.9 Hz, 14-CH), 7.47 (d, 1 H, *J* = 6.9 Hz, 11-CH)

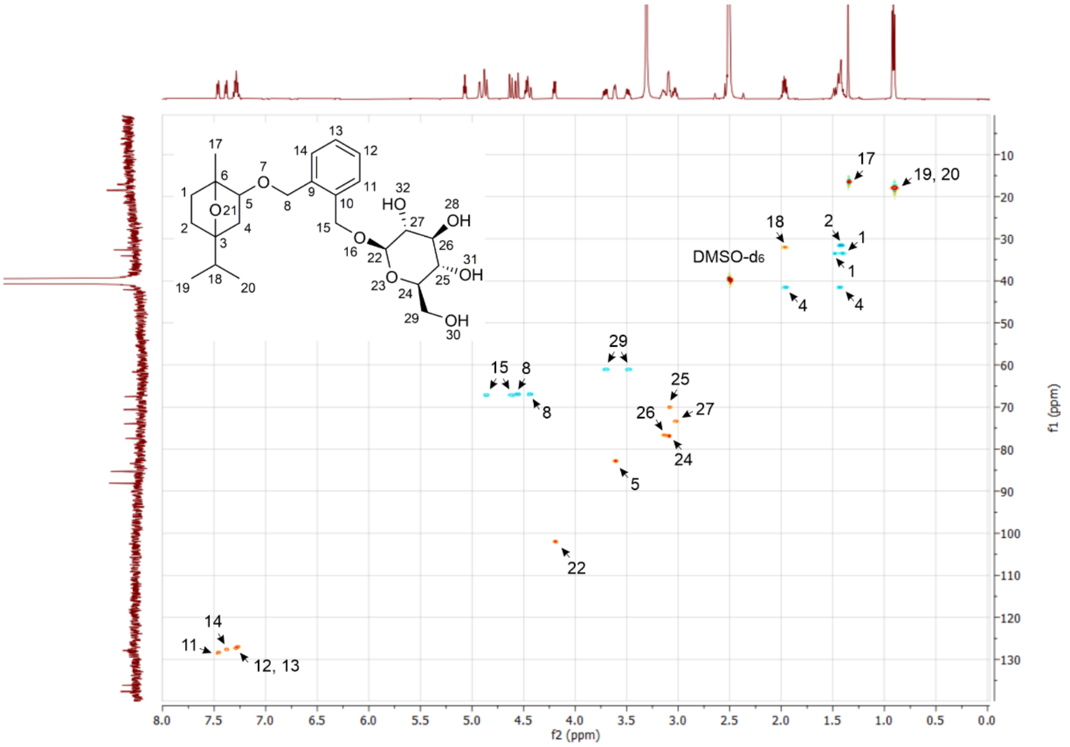

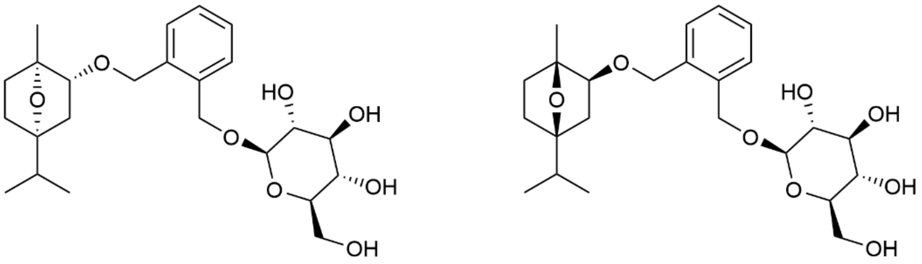

(3*R*, 5*R*, 6*S*) form (3*S*, 5*S*, 6*R*) form

Supporting Figure S3. 2D-HSQC spectrum of 15-hydroxy cinmethylin β-D-glucoside produced by *C. tinctorius* UGT71E5 is shown. DMSO-d_6 was used as solvent. Diastereomeric structures of 15-hydroxy cinmethylin β-D-glucoside are shown on the bottom.

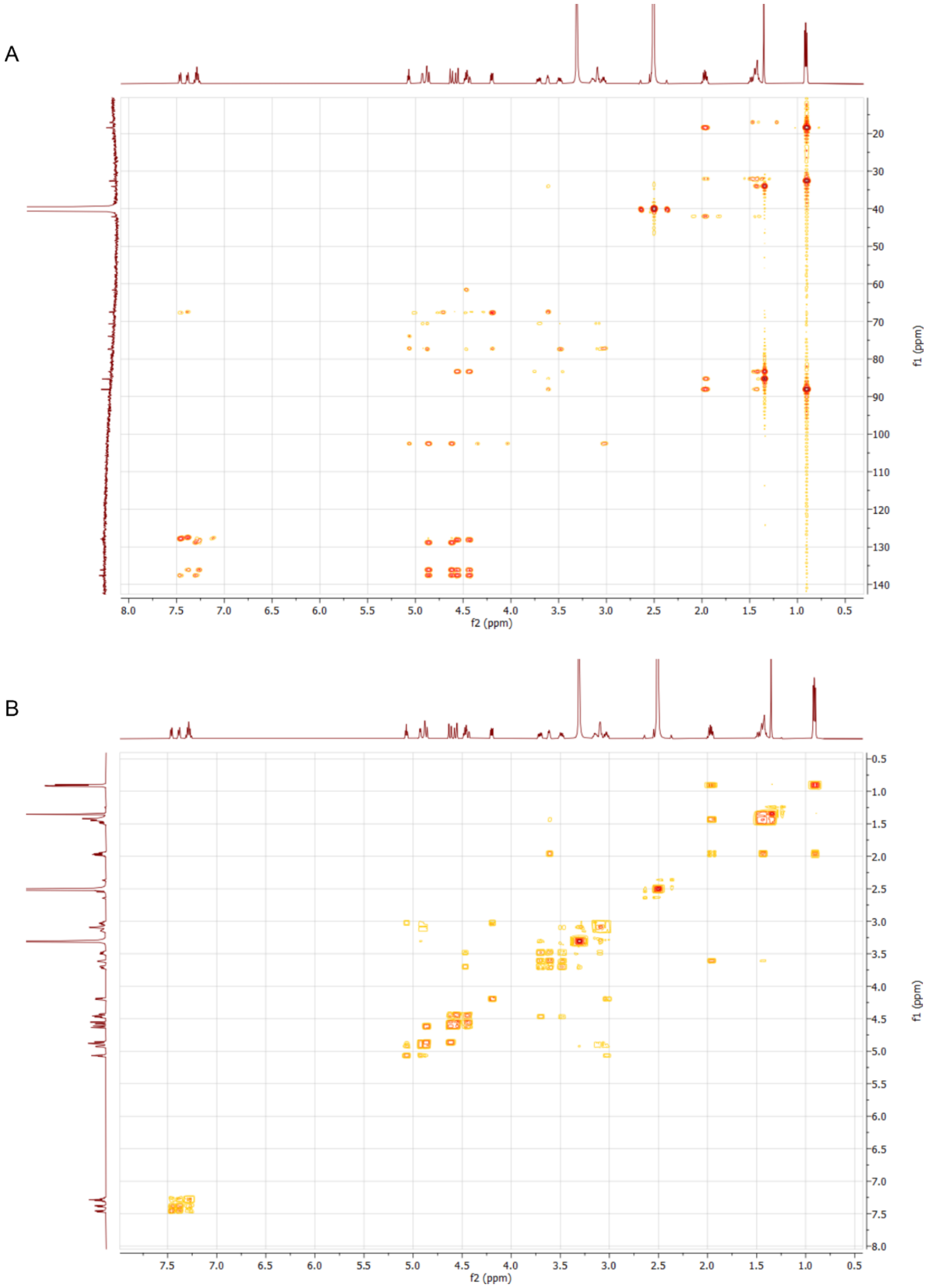

Supporting Figure S4. 2D-HMBC and COSY spectra of 15-hydroxy cinmethylin β-D-glucoside produced by *C. tinctorius* UGT71E5 are shown. 2D-HMBC spectrum is shown in panel A. COSY spectrum is shown in panel B. DMSO-d_6 was used as solvent.

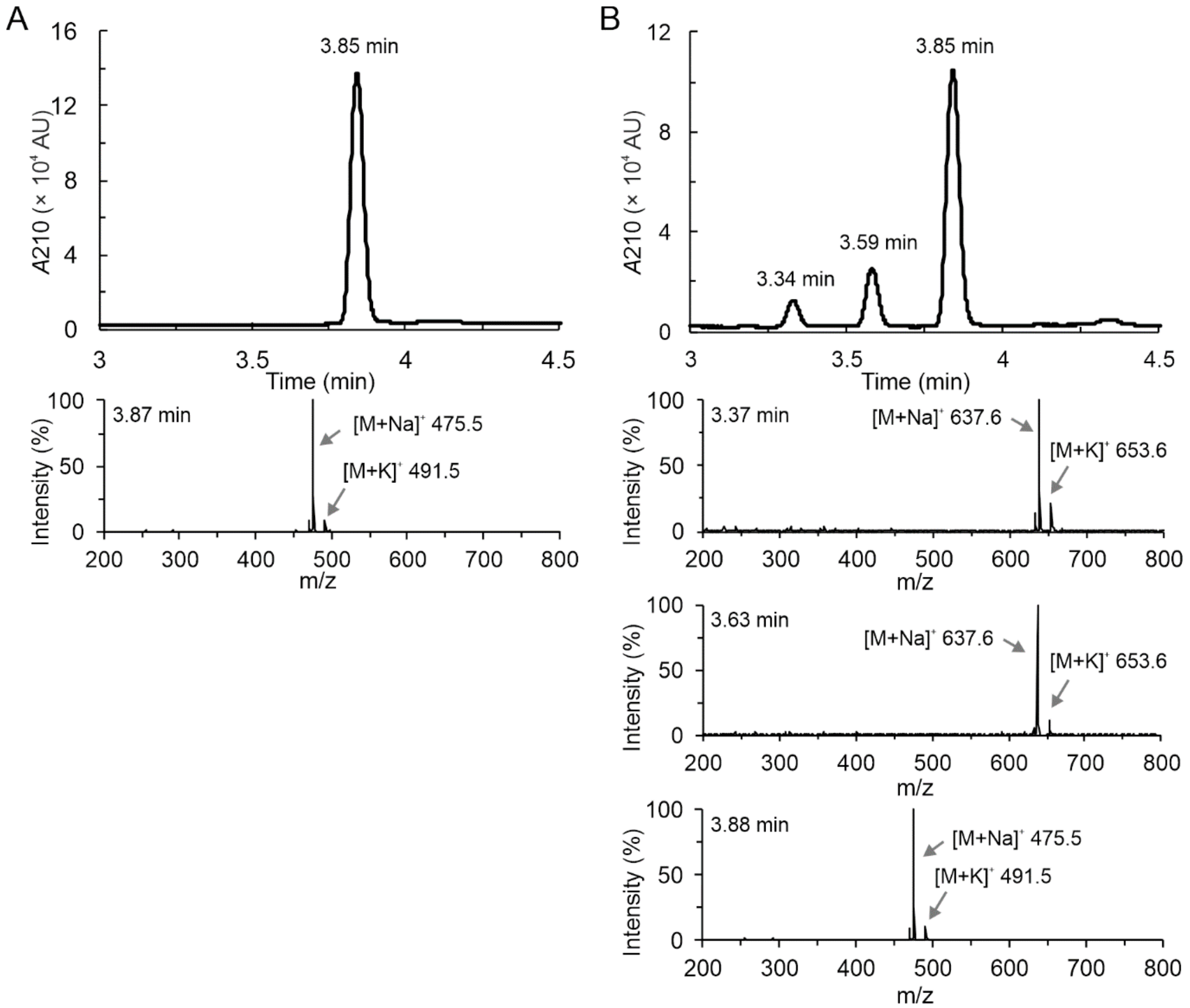

Supporting Figure S5. Mass analysis of 15-hydroxy cinmethylin β-D-glucoside synthesized enzymatically (A) and bis-glycosides formed by *Bc*GT1 after incubation at 37 °C for 22 h (B) is shown. Mono- and di-glucosylated products of 15-hydroxy cinmethylin were detected at 210 nm (top). 15-Hydroxy cinmethylin β-D-glucoside (mass = 452.5; Na^+, 475.5; K^+, 491.5) was eluted at 3.85 min. The putative disaccharide glycosides of 15-hydroxy cinmethylin (mass = 614.7; Na^+, 637.6; K^+, 653.6) were observed at 3.34 min and 3.59 min. Mass peaks were shifted about 0.02-0.05 min (bottom).

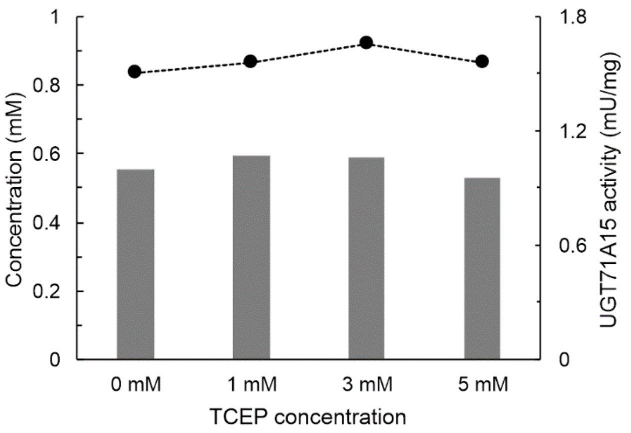

Supporting Figure S6. Effect of TCEP concentration on glycosylation of 15-hydroxy cinmethylin by UGT71A15 is shown. The enzyme solution (3 mg/mL) was prepared in HEPES buffer (100 mM, pH 8.0) additionally containing KCl (100 mM) and $MgCl_2$ (13 mM). Initial concentration of 15-hydroxy cinmethylin was 1 mM. DMSO (4%, v/v) was used as co-solvent. UDP-glucose was 2 mM. Reducing agent, tris(2-carboxyethyl)phosphine (TCEP), was used up to 5 mM. Total volume was 0.3 mL. The reaction was conducted at 30 °C. Samples were taken at 0, 1, 4, 6, and 22.5h. Glycosylation activities of UGT71A15 (closed circles) were obtained by the formation of 15-hydroxy cinmethylin β-D-glucoside for 1 h. The yields of 15-hydroxy cinmethylin β-D-glucoside for 22.5h (gray bars) are shown.

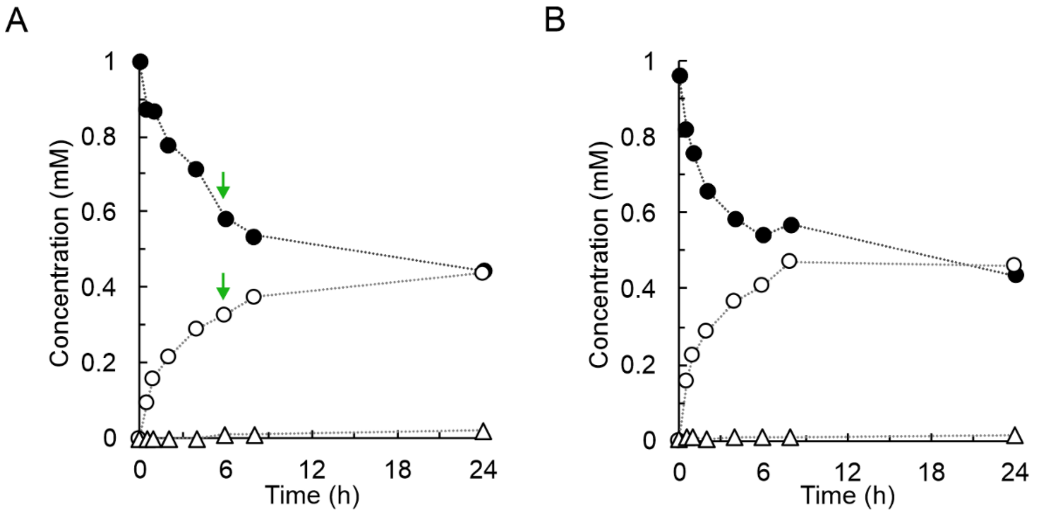

Supporting Figure S7. Effect of enzyme amounts on glycosylation of 15-hydroxy cinmethylin with UGT71A15 is shown. HEPES buffer (100 mM, pH 8.0) containing KCl (100 mM) and $MgCl_2$ (13 mM) was used. 15-Hydroxy cinmethylin (closed circles, 1 mM) was used. UDP-glucose concentration was 2 mM. DMSO (4%, v/v) was used. Total volume was 0.3 mL. Temperature was 30 °C. 15-hydroxy cinmethylin β-D-glucoside (open circles) and the putative disaccharide glycosides of 15-hydroxy cinmethylin (open triangles) were formed. (A) UGT71A15 (3 mg/mL) was added at 0 h and 6 h (green arrow). (B) Initial concentration of UGT71A15 was 6 mg/mL.

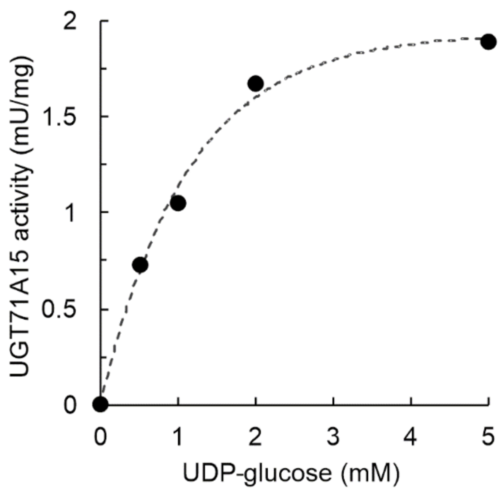

Supporting Figure S8. Effect of UDP-glucose concentration on the formation of 15-hydroxy cinmethylin β-D-glucoside by UGT71A15 is shown. HEPES buffer (100 mM, pH 8.0) containing KCl (100 mM) and $MgCl_2$ (13 mM) was used. UGT71A15 (3 mg/mL) was incubated with 15-hydroxy cinmethylin (1 mM) and UDP-glucose (up to 5 mM) at 30 °C. DMSO (4%, v/v) was used. Total volume was 0.3 mL. Glycosylation activities of UGT71A15 (closed circles) were obtained by the formation of 15-hydroxy cinmethylin β-D-glucoside for 1 h. V_{max} was 2.3 mU/mg and half of V_{max} was observed with 1.1 mM UDP-glucose (double exponential fit, $R^2 = 0.9937$, gray dotted line).

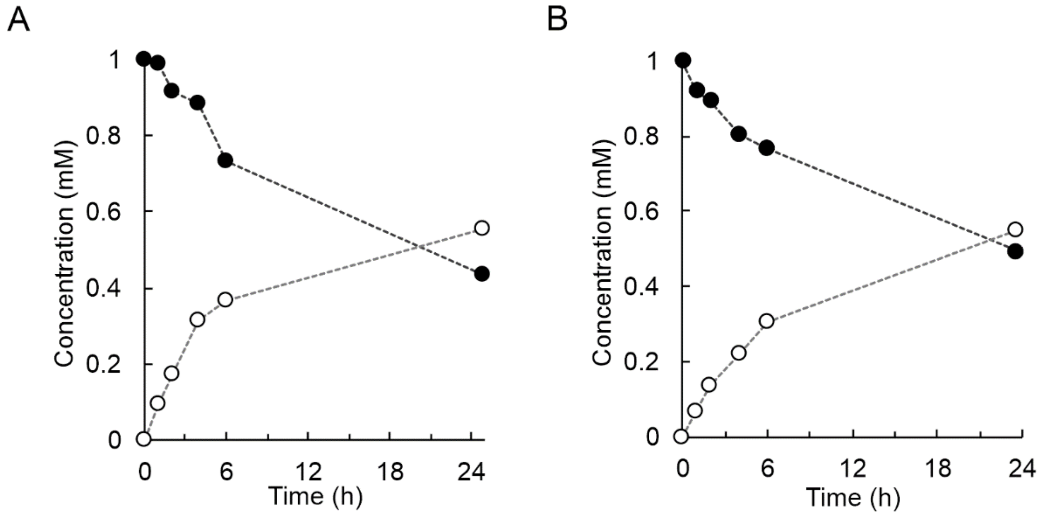

Supporting Figure S9. Two-enzyme cascade glycosylation of 15-hydroxy cinmethylin by UGT71A15 and *Gm*Susy is shown. HEPES buffer (50 mM, pH 7.0) containing KCl (50 mM) and $MgCl_2$ (13 mM) was used. UGT71A15 (3 mg/mL) was incubated with 15-hydroxy cinmethylin (1 mM) at 30 °C. DMSO (4%, v/v) was used. Sucrose (100 mM) and *Gm*Susy (0.05 mg/mL, 0.20 U) were used for donor recycling. UDP concentration was 1 mM (A) and 2 mM (B). Total volume was 0.3 mL. Closed and open circles indicate 15-hydroxy cinmethylin and its β-D-glucoside, respectively.

References

(1) Chiu, H.-H.; Shen, M.-Y.; Liu, Y.-T.; Fu, Y.-L.; Chiu, Y.-A.; Chen, Y.-H.; Huang, C.-P.; Li, Y.-K. Diversity of sugar acceptor of glycosyltransferase 1 from *Bacillus cereus* and its application for glucoside synthesis. *Appl. Microbiol. Biotechnol.* **2016**, *100*, 4459–4471.

(2) Yan, Z.; Caldwell, G. W.; Gauthier, D.; Leo, G. C.; Mei, J.; Ho, C. Y.; Jones, W. J.; Masucci, J. A.; Tuman, R. W.; Galemmo, R. A.; Johnson, D. L. *N*-Glucuronidation of the platelet-derived growth factor receptor tyrosine kinase inhibitor 6,7-(dimethoxy-2,4-dihydronideno[1,2-c]pyrazol-3-yl)-(3-fluoro-phenyl)-amine by human UDP-glucuronosyltransferases. *Drug Metab. Dispos.* **2006**, *34*, 748–755.

(3) Williams, G. J.; Zhang, C.; Thorson, J. S. Expanding the promiscuity of a natural-product glycosyltransferase by directed evolution. *Nat. Chem. Biol.* **2007**, *3*, 657–662.

(4) Xie, K.; Chen, R.; Chen, D.; Li, J.; Wang, R.; Yang, L.; Dai, J. Enzymatic *N* -glycosylation of diverse arylamine aglycones by a promiscuous glycosyltransferase from *Carthamus tinctorius*. *Adv. Synth. Catal.* **2017**, *359*, 603–608.

(5) Lepak, A.; Gutmann, A.; Nidetzky, B. β-Glucosyl fluoride as reverse reaction donor substrate and mechanistic probe of inverting sugar nucleotide-dependent glycosyltransferases. *ACS Catal.* **2018**, *8*, 9148–9153.

(6) Lepak, A.; Gutmann, A.; Kulmer, S. T.; Nidetzky, B. Creating a water-soluble resveratrol-based antioxidant by site-selective enzymatic glucosylation. *ChemBioChem* **2015**, *16*, 1870–1874.
